# Pre-Stroke Frailty and Outcomes following Percutaneous Endoscopic Gastrostomy Tube Insertion

**DOI:** 10.3390/healthcare12161557

**Published:** 2024-08-06

**Authors:** Karan Gupta, Eleanor Williams, Elizabeth A. Warburton, Nicholas Richard Evans

**Affiliations:** 1 Department of Clinical Neurosciences, University of Cambridge, Cambridge CB2 0QQ, UK; kg450@cam.ac.uk (K.G.); eaw23@medschl.cam.ac.uk (E.A.W.); 2 Department of Nutrition and Dietetics, Cambridge University Hospitals NHS Foundation Trust, Cambridge CB2 0QQ, UK; eleanor.williams16@nhs.net

**Keywords:** stroke, frailty, percutaneous endoscopic gastrostomy, post-stroke dysphagia, discharge destination

## Abstract

Background: Frailty is common in stroke, where it exerts disease- and treatment-modifying effects. However, there has been little work evaluating how frailty influences outcomes after percutaneous endoscopic gastrostomy (PEG) tube insertion. This study investigates the relationship between pre-stroke frailty and one-year mortality following PEG insertion. Methods: A pre-stroke frailty index (FI) was calculated for individuals with post-stroke dysphagia who underwent PEG insertion between March 2019 and February 2021. Mortality was recorded at one year, as well as instances of post-PEG pneumonia and discharge destination. Results: Twenty-nine individuals underwent PEG insertion, eleven (37.9%) of whom died in the subsequent year. The mean (SD) FI for those who survived was 0.10 (0.09), compared to 0.27 (0.19) for those who died (*p* = 0.02). This remained significant after adjustment for age and sex, with each 0.1 increase in the FI independently associated with an increased odds of one-year mortality (aOR 1.39, 95% CI 1.17–1.67). There was no association between frailty and post-PEG pneumonia (0.12 (0.21) in those who aspirated versus 0.11 (0.18) in those who did not, *p* = 0.75). Conclusions: Pre-stroke frailty is associated with increased one-year mortality after PEG, a finding that may help inform shared clinical decision-making in complex decisions regarding PEG feeding.

## 1. Introduction

Dysphagia is a common complication following stroke, affecting up to 42% of stroke survivors [1]. The control of swallowing is complex, involving a network of multiple brain regions where stroke may result in sensory impairment, muscle weakness, decreased co-ordination, or impaired reflexes relating to swallowing [2]. The severity of dysphagia can range from difficulty swallowing certain consistencies of food or liquid to a complete inability to swallow, with a consequent risk of aspiration. Many stroke survivors can make significant improvements through rehabilitation, though alternative feeding methods may be required for those with persistent deficits in order to ensure nutritional and hydration needs are met safely. One such method is enteral feeding via the insertion of a percutaneous endoscopic gastrostomy (PEG) tube. Consideration of such feeding approaches requires a multidisciplinary approach and may involve challenging clinical and ethical decisions about efficacy and tolerability, as well as consideration of the individual’s overall prognosis.

Prognostication post-stroke is increasingly guided by explicit consideration of an individual’s pre-stroke frailty. Frailty, the loss of physiological reserve to withstand a stressor event, is related to—but distinct from—age, multimorbidity, and disability [3]. Frailty is common in stroke (with two-thirds of individuals having some degree of frailty [4]) and is associated with a range of disease- and treatment-modifying effects across the stroke pathway, including early mortality [5,6]. However, to date there has been no evaluation of whether pre-stroke frailty is associated with poorer outcomes after PEG tube insertion.

Frailty can be assessed in the clinical setting using a number of different methods. A phenotypic model considers the five main phenotypical characteristics of frailty—weight loss, self-reported exhaustion, low levels of activity, slow gait speed, and weak grip strength—where there is a clear rise in mortality and morbidity with increasing frailty [7]. An alternative is the ‘cumulative deficit model’, which is predicated on the principle that “the more things individuals have wrong with them, the higher the likelihood that they will be frail” [8]. This latter approach provides a sensitive approach to quantifying frailty using existing diagnoses and clinical coding [9,10,11].

This study investigates the relationship between frailty and outcomes post-PEG tube insertion, specifically the associations with post-PEG pneumonia and one-year mortality following the procedure, in order to guide prognostication and clinical decision-making.

## 2. Materials and Methods

### 2.1. Patient Cohort and Setting

This case–control study considered all individuals with persistent post-stroke dysphagia necessitating PEG tube insertion at our centre between March 2019 and February 2021. Our centre is a tertiary neurosciences centre covering an urban and surrounding semi-rural population of around 600,000 individuals, admitting approximately 850 strokes per year. All individuals in this study underwent post-stroke neurorehabilitation, receiving care from a multidisciplinary team incorporating medical, nursing, physiotherapy, occupational therapy, neuropsychology, dietician, and speech and language therapy input. Individuals with sustained dysphagia underwent swallow assessment with either videofluoroscopy or fiberoptic endoscopic evaluation of swallowing (FEES) to guide inpatient rehabilitation of the swallow and clinical management. Individuals with persistent significant post-stroke dysphagia were considered for PEG tube insertion at a Feeding Issues Multidisciplinary Team Meeting involving stroke physicians, gastroenterologists, geriatricians, palliative care physicians, dieticians, and specialist nurses in enteral feeding.

Demographic details (age, sex) and co-morbidities were collected prospectively in routine clinical practice, and extracted retrospectively for analysis by KG from electronic medical records and blinded to outcome data. Stroke-specific details (type of stroke and severity) were also collected prospectively at the time of stroke but extracted retrospectively from electronic medical records and data from the Sentinel Stroke National Audit Programme (SSNAP), a national audit of all strokes admitted to comprehensive and acute stroke centres across the United Kingdom. Stroke severity was measured using the National Institutes of Health Stroke Severity (NIHSS) scale, which is a standardised tool used to assess the severity of neurological deficit following stroke (where scores of 1–4 typically denote mild stroke, 5–15 moderate stroke severity, 16–20 moderate to severe stroke, and above 20 indicating a severe stroke deficit). In instances where individuals with ischaemic stroke received thrombolytic therapy or mechanical thrombectomy, the NIHSS measured at 24 h after intervention was used as a measure of the severity of the persistent neurological deficit.

### 2.2. Assessment of Pre-Stroke Frailty

Components of the frailty index were collected prospectively at the time of admission relating to the individual’s status two weeks prior to admission. Pre-stroke frailty was calculated retrospectively from these data (blinded to outcome) using a validated frailty index (FI) (Table 1) [12]. Biochemical results were taken from blood tests at the time of admission. This cumulative deficit approach to assessing frailty involves 33 deficits (including co-morbidities, functional performance, and biochemical results) against which the individual is scored, producing an index between 0 and 1 where a higher score indicates more severe frailty [13,14]. A threshold of between 0.21 and 0.25 is used most frequently to distinguish between frail and non-frail individuals, though its use as a continuous variable (as opposed to a dichotomised measure) is generally recommended [15]. Consequently, our study considered both continuous and dichotomised measures of frailty (using an FI of ≥0.25 as a cut-off, as identified by Rockwood et al. [16]).

For the purposes of this study, the individual’s FI was calculated for the two-week period prior to their stroke. Data were collected retrospectively from electronic medical records—incorporating medical, nursing, therapy, dietician, and pharmacy assessments of pre-stroke status—by KG, blinded to outcomes.

### 2.3. Clinical Outcomes

Clinical outcomes were measured retrospectively from electronic case records and considered mortality at one-year post-PEG tube insertion (primary outcome). Secondary outcomes included the incidence of aspiration pneumonia within 30 days of PEG tube insertion (considered as instances where there were clinical signs of aspiration pneumonia supported by radiological and/or biochemical evidence of infection) and discharge destination (considered as discharge to an in-patient rehabilitation facility, own home, institutional care (residential/nursing home), or end of life care).

### 2.4. Statistical Analysis

Statistical analysis was conducted using R (version 4.1.1, The R Foundation). Data were tested for normality using Shapiro–Wilk testing. Groups were compared using Mann–Whitney U testing (non-parametric data), *t*-testing (parametric data), or the Z test for two population proportions. Multivariable analyses included all variables from the univariable analysis with refinement via stepwise regression using backwards elimination determined by the Akaike information criterion method. Interactions between these variables were considered, with only the interaction between age and sex reaching statistical significance and subsequently being included in the multivariable modelling. Assumptions for logistic modelling were tested (through review of residuals) and fulfilled.

## 3. Results

### 3.1. Patient Cohort

Twenty-nine individuals had a PEG tube inserted during the study period. Seventeen (58.6%) individuals had had ischaemic strokes and twelve (41.4%) haemorrhagic strokes. Twenty-six (89.7%) were independent at home prior to their stroke, two (6.9%) were in residential homes, and one (3.4%) was in a nursing home.

The median time from stroke to PEG tube insertion was 63 (IQR 53–74) days.

### 3.2. One-Year Mortality

Eleven (37.9%) had died by one year following PEG tube insertion. When dichotomising by frail/non-frail status, eight (27.6%) of the cohort were frail. Clinical characteristics for patients are shown in Table 2.

For those who died, the median time from PEG tube insertion to death was 148 (interquartile range 66–251) days. Clinical characteristics for the two cohorts (death/survival at one-year post-PEG tube insertion) are shown in Table 3.

Frailty remained independently associated with one-year mortality after adjustment for age and sex, with an adjusted r^2^ of 0.62 (Table 4).

### 3.3. Aspiration

Of the 29 individuals, 19 (65.5%) were treated for aspiration within 30 days of PEG tube insertion. There was no significant difference in any of the clinical characteristics between those who aspirated within 30 days of PEG insertion and those who did not (Table 5).

### 3.4. Discharge Destination

The median time from PEG tube insertion to discharge was 29.5 (IQR 15.5–49.5) days. Of the twenty-nine individuals, six (20.7%) were discharged to their own home, nine (31.0%) were transferred to an inpatient rehabilitation facility, twelve (41.4%) were discharged to institutional care (three of whom had been in institutional care prior to their stroke), and two (6.9%) received end of life care (both of whom were previously living at home).

**Figure 1:**
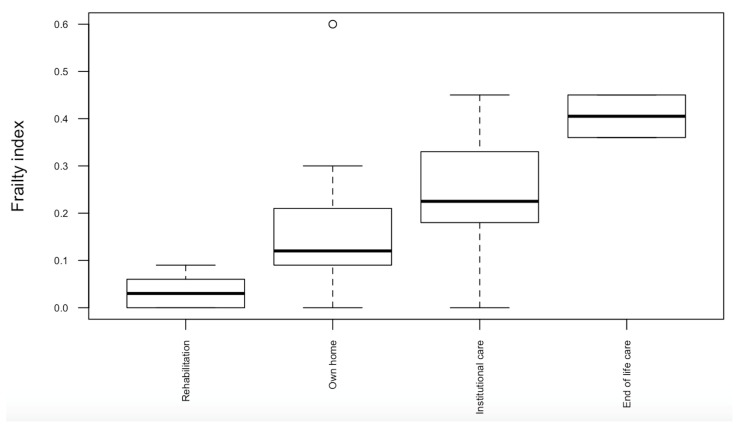
Boxplot of pre-stroke frailty indices according to discharge destination for stroke survivors post-PEG tube insertion.

Figure 1 shows the distribution of frailty indices for stroke survivors being discharged to different settings.

Stroke survivors with a PEG being discharged to institutional care facilities had higher levels of pre-stroke frailty than those who were discharged to their own home or further in-patient rehabilitation (median FIs 0.23 (0.18–0.33) versus 0.06 (0.03–0.12), respectively, *p* = 0.02).

Of the 11 individuals who died in the year following PEG tube insertion, none were discharged to their home environment (1 died in an in-patient rehabilitation facility and 10 died in either institutional care or hospice care). In contrast, amongst the eighteen who survived, only four were discharged to institutional care (with seven being discharged home and seven discharged to in-patient rehabilitation).

## 4. Discussion

In this small observational study, we have found that pre-stroke frailty was associated with increased one-year mortality after PEG but not with post-PEG aspiration. Decisions regarding PEG insertion can be complex as clinicians also have to consider the severity of dysphagia, nutritional requirements, reversibility of the condition, patient preference, risk of aspiration, ability to withstand the procedure, and overall prognosis and quality of life. Our findings may inform clinical management relating to frequently challenging feeding decisions, where the explicit assessment of frailty may inform prognostication relating to outcomes following PEG insertion.

Similar decisions are seen in other life-limiting conditions. The effect of PEG insertion in advanced dementia has little effect on improving function or nutritional status or extending life [17,18]. Yet despite this, there remains variation between clinicians regarding PEG insertion in advanced dementia and for those approaching end of life [19,20]. Whether frailty generates similar clinical variation remains to be seen.

Previous work considering predictive factors for mortality after post-stroke PEG tube insertion identified that age is independently associated with increased post-procedure complications [21,22]. However, it remains unclear to what extent this finding relates to age directly versus the associated higher levels of frailty in older age groups. In our results, consideration of frailty rendered age non-significant in the multivariable analysis. In contrast, early post-PEG mortality has been reported for individuals with lower serum albumin levels and elevated C-reactive protein [23,24,25]. Such biochemical abnormalities are also associated with a reduced duration of survival following PEG tube insertion [26]. These biochemical findings are commonly associated with frailty phenotypes and hence may be consistent with the findings in our cohort.

To date, there has been little consideration of the impact of frailty itself on clinical trajectories following PEG insertion. Increasing frailty is associated with increased mortality across a range of conditions [27], including stroke [4,6], and the finding of an increased one-year mortality in our cohort is consistent with this. Furthermore, there is also evidence demonstrating an association between pre-stroke frailty and worse outcomes in the general stroke population, such as prolonged length of hospital stay, hospital readmission, quality of life, and attenuated benefit from rehabilitation [5,28]. This raises the possibility that PEG tube insertion for stroke survivors with frailty occurs in the already-established poorer trajectory relating to the frailty, rather than the PEG tube itself having a disease-modifying effect. In other words, although the PEG insertion itself is unlikely to contribute to the mortality in a frailer cohort, recognising that it occurs in the context of frailty as a life-limiting condition will likely help inform discussions and expectations around goals of care relating to feeding decisions.

Such considerations regarding feeding decisions after stroke are multifaceted and complex. It is inappropriate to conclude from our data that the higher mortality amongst frail stroke survivors with severe dysphagia should automatically preclude them being offered PEG tube insertion. Instead, consideration of how pre-morbid frailty affects post-PEG trajectories may provide more information for deciding on the relative merits of undergoing PEG tube insertion versus alternative options (such as “risk feeding” and advance care planning). Ultimately, a shared decision regarding PEG tube insertion is required, and clear prognostication may facilitate the stroke survivor in making decisions regarding intervention. Further work comparing outcomes to those who met the criteria for PEG insertion but opting for palliative approaches, as well as exploring the views of quantity and quality of life amongst stroke survivors, may be advantageous for better understanding such considerations.

Our data show that the time from PEG insertion to death in those who died was several months (median 148 days), through from our data it is not possible to say whether PEG tube insertion and subsequent feeding increased time to death amongst frailer stroke survivors, though it is likely. For some (both stroke survivors and clinicians), PEG may be considered an appropriate means to maintain nutritional support and hydration during end of life care, whilst for others the poorer prognosis for pre-morbidly frail individuals may mean they favour conservative approaches that minimise medical intervention during this period. Our results indicate that there was not an association between pre-stroke frailty and aspiration following PEG tube insertion, but it does not cover other factors in the periprocedural period that may have implications on quality of life.

Furthermore, an important consideration is that insertion of a PEG tube may facilitate discharge from hospital, which may have resource and patient preference implications. The lack of a definitive feeding route frequently limits the ability to discharge an individual either to their own home, further in-patient rehabilitation, or institutional care, as typically these facilities are unable to manage feeding via nasogastric tubes. Hence, for stroke survivors with concurrent frailty who are not actively dying but have a life-limited prognosis, PEG tube insertion may provide the means by which they can be discharged to receive palliative care in a non-hospital environment. Such interventions may have important implications for quality of life in end of life care for stroke survivors with frailty.

In our study, the median time from stroke to PEG insertion was 63 days. Several studies have compared early PEG insertion (within 1–2 weeks of stroke) with later PEG insertion (beyond two weeks after stroke), finding early PEG insertion was associated with shorter length of hospital stay and a higher likelihood of discharge home or to acute rehabilitation for individuals aged below 85 years (above this there was no significant difference in terms of discharge destination between early versus late insertion). However, there was no independent association between timing of PEG insertion and mortality [29]. A similar finding that the timing of PEG insertion after ischemic stroke did not impact short-term mortality or complications has also been reported in a cohort of 161 stroke survivors where the median age was 66 years [30], an age profile similar to our cohort. In contrast, in a mixed sample of ischemic and haemorrhagic stroke, early PEG insertion within seven days of stroke was associated with increased 30-day mortality, with a reduction in 30-day mortality for each additional week delayed. However, this association was not seen with mortality at six months [31]. Consequently, although the median time from stroke to PEG insertion in our cohort was significantly longer than these early time windows, the results of these studies suggest that the timing of PEG insertion relative to stroke is unlikely to have affected one-year mortality. This is supported by the results of our multivariable analysis.

Our cohort considered all strokes, regardless of type, and included a relatively high number of haemorrhagic strokes (11 haemorrhagic strokes, 37.9%) compared to the approximate one in five seen in clinical practise. This larger proportion in our sample may be due to the small sample size but conflicts with the findings of a large study of UK stroke survivors that found that individuals with haemorrhagic stroke were less likely to receive PEG compared to ischemic stroke survivors [32]. This may be due to more frequent (though not statistically significant) swallow recovery in intracerebral haemorrhage noted in one study [33]. Our results showed a higher, but not statistically significant, proportion of individuals with haemorrhagic stroke in the group that survived to one year compared to those that died, but this variable was eliminated from multivariable analysis when considered alongside stroke severity during model optimisation. Similarly, there was no significant difference between the proportion of haemorrhagic strokes between stroke survivors who aspirated after PEG and those who did not. Although no modulating effect was seen with the type of stroke in our data, the small sample is likely underpowered to detect this, and hence future work should include stroke type and aetiology as co-variables. Furthermore, the impact of the type of stroke on outcomes after PEG insertion is poorly understood and represents an important avenue of future research.

The finding that pre-stroke frailty was not associated with the development of pneumonia following PEG insertion is an interesting one. In the general population, frailty is associated with an increased vulnerability to infections, including healthcare-associated infections relevant to our cohort being cared for in an institutional setting [34]. In particular, frailty has been found to be associated with the development of pneumonia due to a combination of likely mechanisms (including the strength of muscles involved in swallowing, reduced oral hygiene, and immunomodulation seen in frailty) [35]. The lack of an association in our study may be due to several factors. Firstly, the small sample size may limit the power to detect an association, particularly as the majority of our cohort had signs of aspiration during their inpatient stay following the procedure, necessitating further investigation in a larger study. Secondly, the development of post-PEG pneumonia may be more influenced by frailty status after the stroke (and/or post-PEG insertion) rather than the pre-stroke frailty status, given the long time interval between stroke and PEG tube insertion in our cohort. Finally, whether the insertion of a PEG tube and subsequent feeding mitigates the effects of pre-stroke frailty when it comes to infection is a possibility, though is less likely given the similar levels of pre-stroke frailty between the groups that did and did not develop pneumonia in our cohort.

Our findings come with caveats. The size of the sample is limited and the findings require replication and validation in a larger cohort. However, the real-world nature of the sample is representative of the population typically receiving enteral feeding in terms of stroke type and severity, as well as pre-morbid status. Our study does not include a comparison against stroke survivors with severe dysphagia where PEG insertion was considered but not undertaken, though arguably such a group would not be appropriate as a control group given that such individuals are unlikely to be directly comparable given the nature of the selection procedure. Furthermore, our study considers only mortality and aspiration, and further work considering the risk of peri-procedural complications, as well as a more detailed assessment of post-procedure function and quality of life measures, would help further inform clinical decision-making. Whilst our pilot study shows an important proof of principle in relation to the treatment-modifying effects of frailty in the cohort of stroke survivors undergoing PEG tube insertion, a future larger prospective multicentre study with longer follow-up is required to definitively establish the associations across the range of outcomes important to clinicians and stroke survivors.

## 5. Conclusions

Decisions regarding long-term enteral feeding in individuals with significant post-stroke dysphagia are complex, requiring input from stroke survivors, families, and all members of the multidisciplinary clinical team. Key to such discussions is a recognition of the individual’s overall clinical trajectory. Our results indicate the potential utility of formal frailty assessment for stroke survivors where PEG tube insertion is being considered in order to inform prognostication and guide clinical decision-making.

## Figures and Tables

**Table 1:** Components of the frailty index [12].

Depression	Haemoglobin (Low)	Liver Disease
Anxiety	Care-home resident	Peptic ulcer
Polypharmacy	Carers	Arthritis
Previous cerebrovascular disease	Sensory impairment (e.g., blindness)	Impaired external ADL
Atrial fibrillation	Hearing aid	Impaired ADL
Diabetes	Bladder incontinence	Mobility aid
Hypertension	Bowel incontinence	Assistance walking
Hyperlipideaemia	Falls	Calcium
Heart failure	Fracture	Albumin (low)
Vascular disease	Cancer	High glucose (>10 mg/L)
Previous myocardial infarction	Chronic obstructive pulmonary disease	Renal failure

ADL: activities of daily living.

**Table 2:** Characteristics of whole cohort, as well as according to dichotomised frailty status.

	All Patients (*n* = 29)	Patients without Frailty (*n* = 21)	Patients with Frailty (*n* = 8)	Significance (between Patients with and without Frailty)
Mean age (SD) (years)	63.8 (20.0)	60.0 (±21.8)	73.6 (±9.5)	*p* = 0.03
Male sex (%)	20 (69.0)	16 (76.2)	4 (50.0)	*p* = 0.17
Smoking (%)	15 (51.7)	10 (47.6)	5 (62.5)	*p* = 0.47
Hypertension (%)	19 (65.5)	12 (57.1)	7 (87.5)	*p* = 0.12
Diabetes (%)	6 (20.7)	3 (14.3)	3 (37.5)	*p* = 0.17
Atrial fibrillation (%)	5 (17.2)	2 (9.5)	3 (37.5)	*p* = 0.08
Mean NIHSS (IQR)	12 (13.0)	18 (3.5)	6 (4)	*p* = 0.13
Haemorrhagic stroke (%)	11 (37.9)	10 (47.6)	1 (12.5)	*p* = 0.08
Median Stroke to PEG interval (days) (IQR)	63 (21.0)	63 (26)	63.5 (17.8)	*p* = 0.77
Mortality (%)	11 (37.9)	5 (23.8)	6 (75.0)	*p* = 0.01

FI: Frailty index, NIHSS: National Institutes of Health Stroke Severity scale.

**Table 3:** Characteristics of cohorts that had survived versus died by one year after PEG tube insertion.

	Survival at One Year Post-PEG Tube Insertion (*n* = 18)	Death by One Year Post-PEG Tube Insertion (*n* = 11)	Significance
Mean age (SD) (years)	59.6 (±23.0)	70.7 (±11.3)	*p* = 0.09
Male sex (%)	14 (77.8)	6 (54.5)	*p* = 0.19
Smoking (%)	8 (44.4)	7 (63.6)	*p* = 0.32
Hypertension (%)	11 (61.1)	8 (72.7)	*p* = 0.52
Diabetes (%)	4 (22.2)	2 (18.2)	*p* = 0.79
Atrial fibrillation (%)	2 (0.11)	3 (27.3)	*p* = 0.26
Mean NIHSS (SD)	13.5 (±8.3)	10.3 (±5.2)	*p* = 0.43
Haemorrhagic stroke (%)	9 (50)	2 (18.2)	*p* = 0.09
Median Stroke to PEG interval (days) (IQR)	65.5 (22.75)	53 (20)	*p* = 0.01
Mean FI (SD)	0.1 (±0.09)	0.27 (±0.19)	*p* = 0.02

FI: Frailty index, NIHSS: National Institutes of Health Stroke Severity scale.

**Table 4:** Multivariable logistic regression for one-year mortality.

	Adjusted Odds Ratio	Significance
FI (per 0.1 increase)	1.39 (1.17–1.67)	*p* = 0.04
Age	1.02 (0.85–1.22)	*p* = 0.29
Male sex	0.71 (0.42–1.22)	*p* = 0.10
NIHSS	1.01 (0.98–1.05)	*p* = 0.51
Smoking	0.85 (0.51–1.42)	*p* = 0.21
Diabetes mellitus	0.81 (0.44–1.47)	*p* = 0.52
Hypertension	0.70 (0.35–1.40)	*p* = 0.37
Stroke to PEG interval	1.00 (1.00–1.00)	*p* = 0.70

FI: Frailty index, NIHSS: National Institutes of Health Stroke Scale.

**Table 5:** Characteristics for cohorts who did not aspirate and who aspirated within 30 days of PEG tube insertion.

	No Aspiration (*n* = 10)	Aspiration (*n* = 19)	Significance
Mean age (SD) (years)	70.7 (±14.4)	60.2 (±21.8)	*p* = 0.13
Male sex (%)	8 (80)	13 (68.4)	*p* = 0.51
Smoking (%)	5 (50)	10 (52.6)	*p* = 0.90
Hypertension (%)	6 (60)	13 (68.4)	*p* = 0.65
Diabetes (%)	2 (20)	4 (21.1)	*p* = 0.94
Atrial fibrillation (%)	2 (20)	3 (15.8)	*p* = 0.77
Mean NIHSS (SD)	9.8 (±7.8)	13.4 (±5.62)	*p* = 0.37
Haemorrhagic stroke (%)	3 (30)	8 (42.1)	*p* = 0.36
Median stroke to PEG interval (days) (IQR)	64.5 (24)	59 (28)	*p* = 0.10
Median FI (IQR)	0.11 (0.18)	0.12 (0.21)	*p* = 0.75

FI: Frailty index, NIHSS: National Institutes of Health Stroke Severity scale.

## Data Availability

The raw data supporting the conclusions of this article will be made available by the authors on request.
